# Reversal of ochronotic pigmentation in alkaptonuria following nitisinone therapy: Analysis of data from the United Kingdom National Alkaptonuria Centre

**DOI:** 10.1002/jmd2.12137

**Published:** 2020-06-22

**Authors:** Lakshminarayan R. Ranganath, Anna M. Milan, Andrew T. Hughes, Milad Khedr, Andrew S. Davison, Peter J. Wilson, Jane P. Dillon, Elizabeth West, James A. Gallagher

**Affiliations:** ^1^ Department of Clinical Biochemistry and Metabolic Medicine Royal Liverpool University Hospital Liverpool UK; ^2^ Institute of Ageing and Chronic Disease, Musculoskeletal Biology I, William Henry Duncan Building Liverpool UK; ^3^ Department of ENT Broadgreen Hospital Liverpool UK

**Keywords:** alkaptonuria, nitisinone, homogentisic acid, eye ochronosis, ear ochronosis, ear cartilage biopsy, reversal, natural history

## Abstract

**Background:**

Increased homogentisic acid (HGA) causes ochronosis. Nitisinone decreases HGA. The aim was to study the effect of nitisinone on the ochronosis progression.

**Methods:**

Photographs of the eyes and ears were acquired from patients attending the National Alkaptonuria Centre (NAC) at V‐1 (pre‐baseline visit), V0 (baseline visit when 2 mg nitisinone was commenced), and yearly at V1, V2, and V3 visits. Photographs were inspected for evolution of ochronotic pigment and also scored categorically to derive eye, ear, and combined ochronosis scores. An ear cartilage biopsy was also carried out at V0 and one year after V3 (V4) and ochronotic pigment was assessed and quantitated. Visits were compared for changes in pigment. Fasting blood and 24‐hour urine samples were collected for measurement of HGA.

**Results:**

There were 80 AKU patients at V0, and 52, 47, and 40 at V1, V2, and V3 in the group with variable numbers (VAR Group) respectively; 23 patients attended once before V0, in the V‐1 visit. Photographs of patients show increase in eye pigment between V‐1 and V0, followed by decrease post‐nitisinone at V1, V2, and V3. Ear and combined ochronosis semiquantitative scoring showed an increase between V‐1 and V0 (*P* < .01), followed by a decrease at V1, V2, and V3, in the VAR group (*P* < .01). Ochronotic pigment in ear biopsy between V0 and V4 showed a 19.1% decrease (*P* < .05).

**Conclusions:**

Nitisinone decreases HGA and partially reverses ochronosis.

**SYNOPSIS**

Homogentisic acid‐lowering by nitisinone resulted in partial reversal of ochronosis in alkaptonuria for the first time bringing a fresh perspective on the natural history of the disease.

## 1 INTRODUCTION

Genetic deficiency of homogentisate 1,2 dioxygenase (HGD, EC 1.13.11.5) activity, inherited as an autosomal recessive trait, causes alkaptonuria (AKU) (OMIM 203500), resulting in accumulation of homogentisic acid (HGA), leading to increased serum concentrations and increased excretion in the urine from birth (Figure S1).^1^ AKU is a rare multisystem disorder characterized by a delayed but slow progression of features when untreated.^2^, ^3^ The frequency of AKU is around 1 in 250,000 in the general population when not characterized by consanguinity.^4^ The clinical picture is dominated by degenerative disease of the spine and joints leading to significant decrease in mobility and quality of life. While the clinical picture can wax and wane due to intermittent crises, the overall trajectory is one of steady progression. Other features of AKU include aortic valve disease, lithiasis, osteopenic fractures, muscle and tendon ruptures, and arthroplasty.^5^, ^6^

HGA is an intermediary in the normal catabolism of surplus dietary tyrosine and phenylalanine. In the presence of normal HGD activity, it is rapidly metabolized to fumarate and acetoacetate, such that concentrations of HGA in the serum and urine are negligible in those without AKU.^7^ In AKU the accumulating HGA deposits over time as a melanin‐like pigment in connective tissues in the body, leading to the change in the material properties of these tissues, and is the main pathophysiological process namely ochronosis.^8^, ^9^ Stiffened and brittle ochronotic tissues breakdown during use leading to the protean manifestations of AKU.^10^ The appearance of the pigmented tissues during post‐mortem and *ex vivo* studies has led to the idea that the ochronotic pigmentation process is irreversible.

In addition to the extensive ochronosis observed in tissues and organs inside the body, it can also be observed in the eyes and the ears, where it is a major clue drawing attention to the diagnosis of AKU.^11^ Pigmentation can be present in the conjunctiva and the sclera, both nasal and temporal to the cornea. In the ear it is the elastic cartilage of the pinna which pigments. Both the eyes and the ears show pigment easily visible to the discerning examiner by around the age of 30 years. The idea that subclinical ochronosis could antedate visible external ochronosis was confirmed in a non‐interventional cross‐sectional study called SOFIA examining photographs of the eyes and ears and by the use of a minimally invasive ear cartilage biopsy where the tissue was then processed using the sensitive Schmorl's staining technique.^12^ SOFIA confirmed that ochronotic pigment can be present both in the eye and the ear by careful examination of photographs even by the age of 16 years.^12^ The evolution of the pigment in the eye and the ear correlates closely with the development of symptoms and signs of the disease in the spine and joints^12^; this suggests that when ochronosis is detected it is likely to be present throughout the body.

Ochronosis has been studied in a BALB/c *Hgd* ^*−/−*^ mouse.^13^, ^14^ The BALB/c *Hgd* ^*−/−*^ mouse model shows increased plasma HGA levels, and extensive chondrocyte pigmentation in the tibio‐femoral (knee) joint via a modified Schmorl's stain. The BALB/c *Hgd* ^*−/−*^ mice also show that initial chondrocyte pigmentation is in the calcified cartilage, pericellular to begin with, progressing to the intracellular compartment, and showing a linear increase in pigmentation with age. Administration of nitisinone to these mice resulted in complete prevention of ochronotic pigment formation when started from birth; delayed administration of nitisinone arrested further ochronotic pigment progression. These data have further reinforced the idea that ochronosis may be irreversible.

Until recently, management of AKU has been largely supportive and palliative lacking an HGA‐lowering therapy.^6^ Nitisinone, a potent inhibitor of hydroxyphenylpyruvate dioxygenase (HPPD, EC 1.13.11.27) activity, developed as a herbicide, has been in use for another fatal tyrosine pathway disorder hereditary tyrosinaemia 1 (HT‐1, OMIM 276700) since 1991.^15^, ^16^ It was postulated that inhibition of HPPD activity could decrease the formation of HGA in AKU in 1998.^17^ Nitisinone has since undergone further development in treatment of AKU both in the National Institutes of Health,^4^, ^18^, ^19^ as well as in the United Kingdom National Alkaptonuria Centre (NAC).^20^, ^21^ It has been possible to closely observe the effect of nitisinone in AKU in the NAC not just on the metabolism but also on the disease process namely ochronosis. Nitisinone therapy in the NAC, and in the NIH, decreased serum and urine HGA by over 95%, offering for all practical purposes a “biochemical” cure of AKU.^19^, ^20^, ^21^, ^22^

The aim of the study discussed further in this manuscript was to examine the effect of nitisinone on the ochronotic process in photographs of the eyes and ears, as well as in ear cartilage biopsies taken four years apart, in patients attending the NAC.

## 2 SUBJECTS AND METHODS

Highly Specialised Services, NHS England (HSS) designated the Royal Liverpool University Hospital to host the NAC. All the NAC data presented here were collected as part of annual audit, a prerequisite to continued funding by HSS, and approved by the lead author's institutional audit committee (Audit no. ACO3836). Data from patients attending the NAC annually over three years of nitisinone therapy is presented; visits were designated V0 (baseline), and V1, V2, and V3 indicative of one, two and three years of nitisinone therapy. Since varying numbers attended yearly visits in this analysis, with new patients enrolling in the NAC on an ongoing basis, the data is labeled as the VAR group (or VAR; variable number of patients at each visit). Nitisinone 2 mg oral daily, used off‐license after approval by HSS, was commenced at V0. Systematic assessments were carried out at all visits in a protocolized manner, as agreed with HSS in order to collect high quality data (Figure S2).

A group of NAC patients also attended a pre‐baseline visit, an extra visit without receiving nitisinone prior to the baseline visit (V‐1); within this group, a subgroup attended V‐1, V0, V1, V2, and V3 visits constituting the SAME Group. As in the VAR group, change in scores between V‐1 and V0) represents follow‐up without nitisinone; change in scores between V0 and V1, V0 and V2, and V0 and V3, represents one, two and three years of nitisinone therapy. Change in scores per month was calculated by dividing score change between visits by the duration between visits in months (Figure S2). For example, V0 – V‐1/duration between visits in months = change in score/month. This change in score/month lends itself for comparison between visits even when duration between visits vary.

### 2.1 Off‐label nitisinone usage

On day 3 of the patient visit V0 to the NAC, nitisinone 2 mg oral was commenced. Fasting serum and 24 hours urine were thereafter collected at V1, V2, and V3. Elective assessments were carried out at each visit to the NAC.

### 2.2 Assessments

Photographs of the eyes and ears were taken at each visit, under standardized conditions in the Medical Photography Department of the hospital. These photographs were visually inspected for ochronotic pigmentation. The photographs of eyes and ears were also scored semiquantitatively for ochronosis, as previously described^20^, ^21^ (Table S1, Figure S3). The photographs were scored blind and by more than one observer, with excellent correlation between the two observers.

Ear cartilage biopsy: A minimally invasive ear cartilage biopsy was carried out at V0 and one year after V3 (V4). The first ear biopsy was carried out on the ear opposite to the sleeping posture adopted; for example, if patient slept on right side, the left ear was sampled at V0; the opposite ear cartilage was sampled at V4. A 4 mm diameter, 1 to 2 mm thick biopsy was taken from the conchal bowl of the ear and stored using a standardized protocol. Each biopsy contained a disc of cartilage 4 mm in diameter and 1 to 2 mm thick. The disc was bisected along the diameter and a thin slice of 0.8 mm was taken from the cut face. This sample was examined using an Olympus SZH binocular microscope in darkfield mode at ×7.5 magnification. The biopsy section was photographed using a 9M pixels DCM 900 camera and images stored as TIFF files. TIFFs were opened in Image J as 8‐bit RGB images. An oval region of interest 3 mm long by 1 mm wide was selected and the mean color intensity in the blue channel was quantified on 255 scale, transformed so that white = 0 and black = 255. Following subtraction of the absorbance of non‐ochronotic tissue the percentage absorbance was calculated (non‐ochronotic tissue = 0 and completely ochronotic tissue = 100). Presence of ochronotic pigmentation was confirmed by histology on serial sections followed by Schmorl staining and microscopy, as described previously.^12^

Consent from patients was obtained separately for the photographs and for the ear cartilage biopsy, as part of the institutional governance system. All patients were provided with an information booklet on the NAC, which stated explicitly that anonymized data would be published and disseminated, as agreed with the funders, Highly Specialised Services, NHS England. All other procedures, including urine and blood sample collection, and data collection were carried out without additional consent, even though the highest standards of ethical care were always followed. The data were collected as part of providing a service and then analyzed as part of ongoing annual audits, rather than as a research study.

### 2.3 Chemical analysis

Samples from 2012 onwards were available for assay. HGA was measured on acidified 24‐hour urine (uHGA_24_) and acidified fasting serum (sHGA) samples from each visit as previously described using liquid chromatography tandem mass spectrometry (LC‐MS/MS) methods.^23^, ^24^

### 2.4 Statistical analysis

Data are presented using a mean and standard deviation (SD). Parametric tests using paired Student's *t* test were employed in order to detect differences in chemical and ochronosis characteristics between AKU visits. Wilcoxon matched‐pairs signed‐ranks test (two‐tail) was used to compare ear cartilage biopsy ochronosis between V0 and V4. Simple linear regressions were the other main analyses generated. Outcome variables were plotted against age and metabolic data (sHGA and uHGA_24_) and regression analyses were carried out. A two‐sided significance level of *P* values < .05 was used throughout. All analyses were conducted using the Graphpad Instat 3 software and figures were generated by the Deltagraph 7 software.

## 3 RESULTS

### 3.1 Demographics and groups

Patients numbers increased year on year in the NAC with continued case‐finding. In the analyses presented in this manuscript, 23 patients attended the NAC twice without receiving nitisinone, the earliest visit was V‐1. 80, 52, 47, and 40 patients attended the NAC at visit V0, V1, V2, and V3 respectively; 52, 47, and 40 pairs of patient data were obtained for one, two and three years of nitisinone therapy respectively for data set comparisons in the VAR group. The age of patients at V‐1, V0, V1, V2, and V3, as well as the duration between visits are shown in Table 1. Of the 23 patient pairs attending V‐1 and V0, 10 also then went on to V1, V2, and V3 visits, meaning that this group had a pre‐nitisinone period and a post‐nitisinone period of follow‐up in the NAC (the SAME group); the mean (SD) age at V‐1 being 47.4 (4.4) years with 6 males and 4 females. The duration between visits in the SAME group is shown in Table 1 (Figure S2). Thirty‐four patients had a minimally invasive ear biopsy taken one year after V3.

**TABLE 1:** Demographic, ochronosis score, and metabolic data in variable (VAR) and same (SAME) groups

VAR group								
	V‐1 vs V0		V0 vs V1		V0 vs V2		V0 vs V3	
	V‐1	V0	V0	V1	V0	V2	V0	V3
Numbers of patient pairs	23		52		47		40	
Age (years)	47.5 ± 14.8	50.8 ± 15.1	47.3 ± 14.7	48.3 ± 14.6	46.7 ± 14.2	48.7 ± 14.1	46.9 ± 14.7	49.9 ± 14.7
Duration between visit	40.5 ± 22.0 months		12.3 ± 0.9 months		24.5 ± 1.3 months		36.8 ± 2.3 months	
Male/female	15M/8F		32M/20F		29M/18F		24M/16F	
	V‐1	V0	V0	V1	V0	V2	V0	V3
Eye scores	8.8 (8.3)	11.4 (9.9)	9.0 (8.1)	8.6 (7.8)	8.0 (7.2)	7.5 (6.8)	8.3 (7.7)	7.7 (7.2)
*P* values	<.01		<.05		<.13		<.09	
Ear scores	3.9 (2.9)	5.2 (2.8)	3.7 (2.8)	3.2 (2.5)	3.6 (2.8)	2.6 (2.5)	4.0 (2.7)	3.0 (2.5)
*P* values	<.01		<.01		<.001		<.001	
Combined scores	12.7 (10.8)	16.6 (11.9)	12.8 (9.8)	11.7 (9.5)	11.6 (8.9)	10.1 (8.6)	12.3 (9.3)	10.7 (9.0)
*P* values	<.0001		<.0001		<.0001		<.001	
sHGA (μmol/L)	26.0 (14.0)	32.8 (18.5)	28.7 (15.4)	6.1 (6.6)	28.2 (15.6)	5.6 (6.6)	26.7 (13.4)	4.5 (6.7)
*P* values	<.73		<.0001		<.0001		<.0001	
uHGA_24_ (mmol/day)	19.1 (16.9)	20.7 (10.2)	20.7 (9.65)	2.62 (0.66)	20.8 (10.0)	2.5 (6.0)	21.6 (10.1)	1.4 (2.0)
*P* values	<.43		<.0001		<.0001		<.0001	

SAME group								
(mean [SD] age at V‐1 was 47.7 (4.4) years; n = 10; 6 male/4 female)								
Duration between visit	36.7 ± 6.9 months		12.8 ± 1.7 months		24.8 ± 1.7 months		36.8 ± 1.7 months	
Eye scores	7.3 (7.5)	8.7 (8.7)	8.7 (8.7)	8.4 (8.3)	8.7 (8.7)	8.3 (8.3)	8.7 (8.7)	8.2 (8.8)
*P* values	<.05		<.56		<.38		<.43	
Ear scores	4.2 (2.7)	5.4 (2.3)	5.4 (2.3)	4.8 (2.3)	5.4 (2.3)	3.8 (2.7)	5.4 (2.3)	4.4 (2.8)
*P* values	<.05		<.28		<.06		<.14	
Combined scores	11.5 (9.9)	14.1 (10.6)	14.1 (10.6)	13.2 (10.3)	14.1 (10.6)	12.1 (10.6)	14.1 (10.6)	12.6 (11.2)
*P* values	<.01		<.1		<.01		<.1	
sHGA (μmol/L)^a^	—	—	33.7 (18.1)	4.3 (1.6)	33.7 (18.1)	3.9 (0.9)	33.7 (18.1)	3.4 (1.0)
*P* values			<.05		<.01		<.001	
uHGA_24_ (mmol/day)^a^	—	—	20.3 (9.6)	0.9 (0.1)	20.3 (9.6)	0.8 (0.7)	20.3 (9.6)	0.9 (0.7)
*P* values			<.001		<.001		<.001	

*Note*: Data shown expressed as mean (SD).

a Lack of data during visit V‐1 in same group. sHGA and uHGA_24_ are expressed as μmol/L and μmol/day respectively.

### 3.2 sHGA and uHGA_24_

All patients had increased urine HGA confirming the diagnosis of AKU at V0. uHGA_24_ concentrations decreased by 78.7% to 96.1% at all follow‐up post‐nitisinone visits compared to V0, for the SAME and the VAR groups (*P* < .01) (Table 1). Similarly, s‐HGA decreased by 78.7% to 89.9% at all post‐nitisinone follow‐up visits as compared to V0, in both the SAME and VAR groups (*P* < .01; Table 1).

### 3.3 Photographs of eyes and ears

**FIGURE 1:**
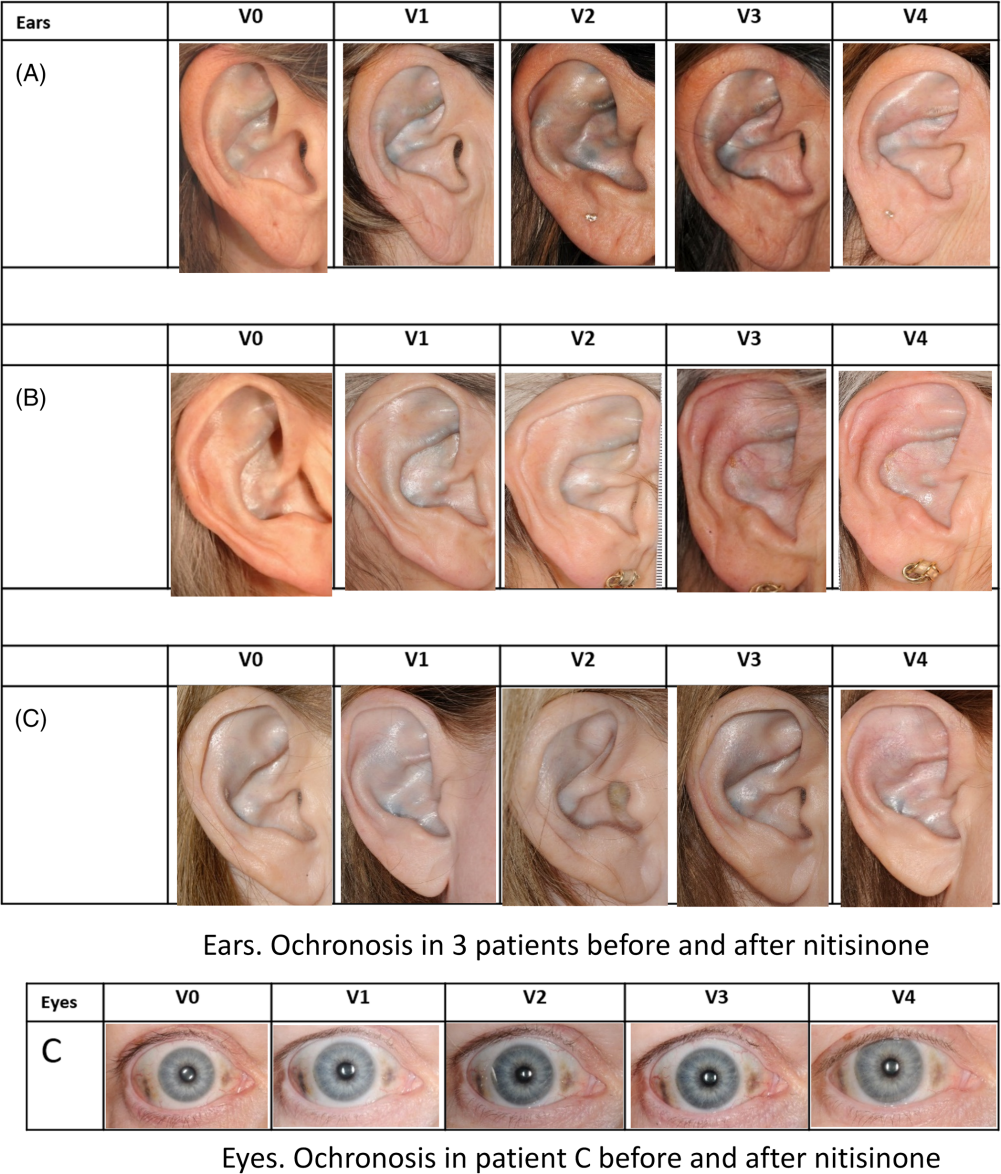
Serial photographs of ochronosis in three patients (A, B, C) in the ears and in the eyes of patient C before and after nitisinone at visits V‐1, V0, V1, V2, and V3

Patients attending the NAC informed the medical personnel that they had noticed a decrease in pigmentation of their ears. Subsequent examination of the photographic images of the ears and the eyes confirmed the impression of patients. All photographs of the eyes and ears post‐nitisinone either showed no increase or some decrease on visual examination. Photographs of the ears of patients A, B and C are shown in Figure 1.X, which shows an apparent increase in pigment between V‐1 and V0, followed by a slow and partial decrease in pigment from V0 until V3. Eye photograph of patient C shows a similar increase between V‐1 and V0 and subsequent decrease from V0 until V3 (Figure 1.Y) It is noteworthy that pigment intensity and shape changes over time.

### 3.4 Ochronosis scores

#### 3.4.1 SAME group

There was a significant increase in eye ochronosis (*P* < .05), ear ochronosis (*P* < .05) and combined ochronosis (*P* < .01) scores between V‐1 and V0, namely pre‐nitisinone. When post‐nitisinone visits (V1, V2, V3) were compared against V0, there was no increase in ochronosis scores (eye, ear or combined), but instead there was a trend of a decrease in scores (Table 1).

#### 3.4.2 VAR group

There was a significant increase in eye ochronosis (*P* < .01), ear ochronosis (*P* < .01) and combined ochronosis (*P* < .0001) scores between V‐1 and V0, namely pre‐nitisinone. When post‐nitisinone visits (V1, V2, V3) were compared against V0, there was a significant decrease in ochronosis scores (ear and combined), with a similar trend for eye scores (Table 1).

### 3.5 Change in scores per month

#### 3.5.1 SAME group score change over time

**FIGURE 2:**
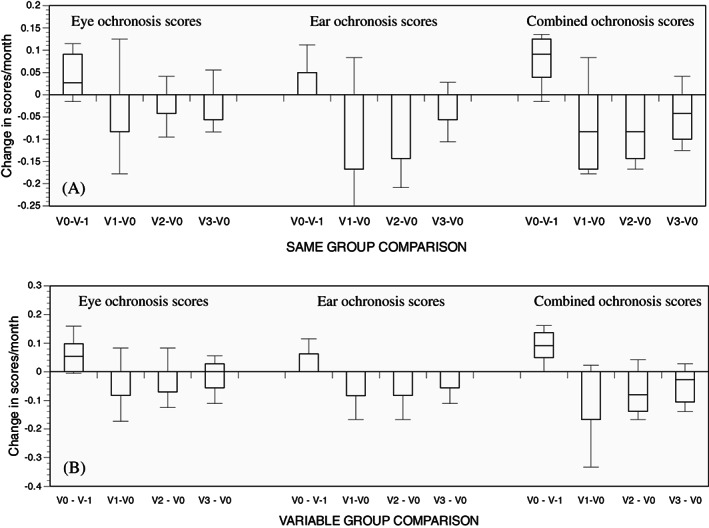
Change in eye, ear, and combined ochronosis scores per month compared across visits. Comparisons made against baseline visit to the NAC (V0), including V0 – V‐1, V1‐V0, V2‐V0, and V3‐V0. Panel A shows SAME group (n = 10) and panel B shows VAR group comparisons. (Box plots with horizontal line (median) inside the box)

The score changes per patient per month showed significant increases in eye ochronosis (*P* < .05), ear ochronosis (*P* < .05) and combined ochronosis (*P* < .01) scores between V‐1 and V0, namely pre‐nitisinone. When post‐nitisinone visits (V1, V2, V3) were compared against V0, there was no increase in ochronosis scores (eye, ear, or combined), and there was a trend to lower scores (Table 1, Figure 2).

#### 3.5.2 VAR group score change over time

The score changes per patient per month showed significant increases in eye ochronosis (*P* < .01), ear ochronosis (*P* < .01) and combined ochronosis (*P* < .0001) scores between V‐1 and V0, namely pre‐nitisinone. When post‐nitisinone visits (V1, V2, V3) were compared against V0, there was a significant decrease in ochronosis scores (ear and combined), with a similar trend for eye scores (Table 1, Figure 2).

#### 3.5.3 Linear regression analysis of baseline ochronosis scores

**FIGURE 3:**
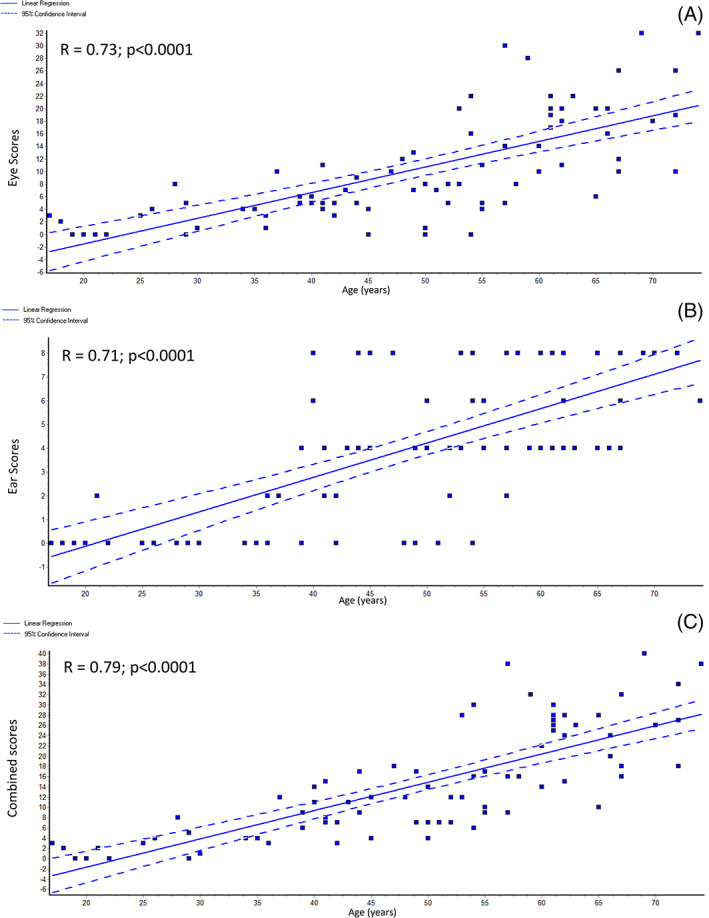
Linear regression graphs of, A, eye; B, ear; and C, combined ochronosis scores against age. *R* refers to correlation coefficient

**FIGURE 4:**
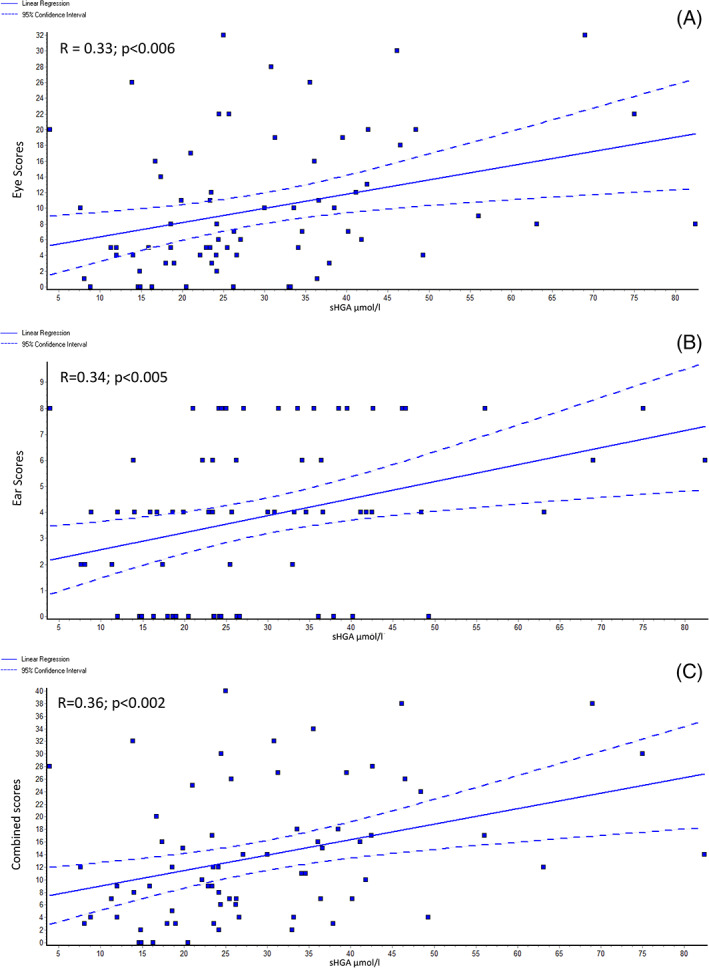
Linear regression graphs of, A, eye ochronosis; B, ear ochronosis; and C, combined ochronosis scores against sHGA at baseline. *R* refers to correlation coefficient. sHGA and uHGA_24_ are expressed as μmol/L and μmol/day respectively

Only V0 (baseline, pre‐nitisinone) were analyzed by linear regression. Eye (*R* = 0.73; *P* < .0001), ear (*R* = 0.71; *P* < .0001), and combined (*R* = 0.79; *P* < .0001) ochronosis scores showed a strong positive relationship with age (Figure 3). Eye (*R* = 0.33; *P* < .01), ear (*R* = 0.34; *P* < .01), and combined (*R* = 0.36; *P* < .01) ochronosis scores also showed a positive relationship with sHGA (Figure 4). Regression analysis between sHGA and age showed a significant positive relationship (*R* = 0.31; *P* < .01); uHGA_24_ and age showed a weak negative trend (*R* = −0.16; *P* < .2) (Figure S4).

#### 3.5.4 Ear cartilage biopsy

**FIGURE 5:**
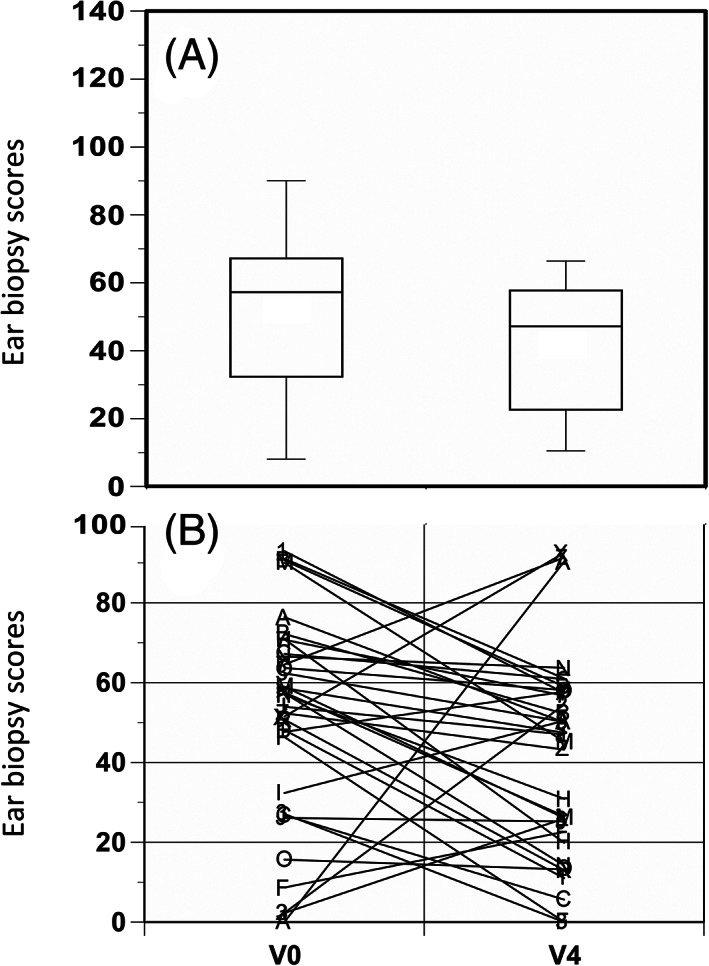
Change in ear biopsy ochronosis scores with mean (SD), A; and individual patient score changes, B, from baseline (V0) to 1 years after V3 (V4). Wilcoxon matched‐pairs signed‐ranks test (two‐tail) showed a *P* < .05

Ear cartilage biopsies at V0 and V4 biopsies were obtained from 34 patients. The results of the individual analyses of V0 and V4 biopsies are shown in Figure 5. Of the 34 paired samples, 26 had a lower score for pigment intensity at V4 compare to V0. Due to the heterogeneity of pigment distribution in the ear, an apparent increase or decrease in pigmentation in individual patient could occur between V0 and V4 samples, however, the mean values for the cohort strongly indicate that there was no overall rise in pigmentation over the treatment period. Instead, there is evidence that pigmentation was significantly reversed by 19.1% (*P* < .05) (Table S2, Figure 5).

## 4 DISCUSSION

Ochronosis was assessed in the analysis presented by three different approaches; the eye discerned changes in pigment qualitatively in different photographs, semiquantitatively by categorical scoring (Table S1, Figure S3), and by quantitative continuous scoring in the ear biopsy. These different approaches were found to be complementary and providing similar conclusions, namely that ochronotic pigment decreased following nitisinone therapy. Ideally, a more continuous measure of assessing pigment in photographs, such as digital quantitation of intensity and area of pigment, should be used, but is not available at the time of this analysis.

In the photographs of the ear, it is noticeable that ochronosis increased without nitisinone, and then decreased after nitisinone, with a suggestion that the intensity of the pigment after three years of nitisinone was less than the prebaseline visit (V‐1). It is also noteworthy that the “reversal” of ochronosis was incomplete over the three years of nitisinone therapy. It remains to be seen if a longer period of nitisinone therapy would reverse the ochronosis more fully. The detection of the change in the ochronosis of the eye required more careful study. It is noteworthy that the intensity and shape of the pigment in the eye (Figure 1) changes between visits pointing to a dynamic rather than a static process. It is worthwhile to remember that the ochronotic pigment in the ear is predominantly found in the hyaline cartilage of the ear. In the eye, it is present both in the superficial looser conjunctival tissue as well as the deeper compact scleral connective tissue.

The advantage of the semiquantitative scoring over the visual inspection of the photographs is the opportunity to carry out statistical analysis on numerical data. Eye, ear and combined eye plus ear scoring showed a similar pattern and intensity of change before and after nitisinone therapy; the combined score analysis not surprisingly was better than the individual eye or the ear scoring. Since the data was collected during the provision of a service the duration between visits showed some variability and in order to adjust for this a change score per month was calculated as already described, and plotted (Figure 2). This clearly shows the change is negative in keeping with decreases in ochronosis, not just arrest, following nitisinone therapy.

The ear cartilage biopsy showed that ochronosis was not uniform throughout the sample and this heterogeneity is a limitation in its usefulness. A non‐invasive analysis is being developed by utilizing Raman Spectroscopy for a future study,^25^ in which a much larger area of the ear tissue could be analyzed. The appearance of ear and eye ochronosis coincides with the appearance of low back and knee pain, suggesting a common evolving ochronotic pathogenetic process.^2^, ^3^

The first ear biopsy was carried out on the ear opposite to the sleeping posture adopted; for example, if patient slept on right side, the left ear was sampled at V0; the opposite ear cartilage was sampled at V4. This was designed to minimize bias as it has been hypothesized that pressure on the ear during sleep could predispose to ochronotic pigment formation; asymmetrical pigmentation in the ear is common.^11^ The ear biopsy conclusions despite the heterogeneity of pigmentation match those of the other two approaches in terms of assessment of ochronosis.

The relatively localized pigmentation of the conjunctivum and sclera could be due to the ultraviolet exposure of these exposed parts of the eyeball. It is noteworthy that Bitot's spots in vitamin A deficiency, pinguecula and pterygium, all arise in the same area where ochronosis in the eye is observed. It has also been hypothesized that eyeball muscle pull could create stresses that subsequently serve as a nidus for ochronotic pigmentation.^11^ Tears like other biological fluids in AKU are likely to contain HGA. However, this has not been shown by chemical analysis; tears form a film over the front of the eye and could also influence pigmentation especially in the conjunctivum.

As previously stated, ochronosis is currently considered to be an irreversible phenomenon. However, as shown in the present data, there is a reversal of ochronosis in the ear and eye following nitisinone. Nothing is known about the potential mechanism of such a change. It is unlikely to be due to a direct nitisinone effect. Since it is the HGA that converts to pigment, and since nitisinone decreases sHGA effectively, it is likely that HGA‐lowering is responsible for the reversal of ochronosis. In support of this, in the present data set, we have shown that there is a significant positive relationship between sHGA and age with ochronosis scores (Figure 4); the possible reason why the relationship between sHGA and ochronosis scores was not as strong as that between ochronosis scores and age may be because the sHGA data used here is a snapshot measurement and does not reflect the lifetime exposure to circulating HGA (Figures 3 and 4). It is likely that pigmentation in AKU is a dynamic process, with formation and removal coexisting. It is also likely that pigment formation is the dominant process when sHGA concentrations are increased, and that a decrease in sHGA allows the pigment removal mechanisms to become dominant. sHGA has been found to increase with age and whether this could be a reason why appearance of overt ochronosis is delayed into the second and third decades is conjectural; removal mechanisms may be masked with increasing sHGA leading to the appearance of visible pigment. Support for this in the current dataset comes from the positive relationship observed between sHGA and age (Figure S4). This idea is emphasized and supported by descriptions of accelerated ochronosis following renal failure and the marked increase in circulating HGA.^26^ The current dataset showed a non‐significant negative relationship between age and uHGA_24_, unlike the relationship between sHGA and age, and is consistent with renal elimination of HGA influencing sHGA.

Precisely how the pigment, which leads to brittle and friable tissue, is mobilized and removed post‐nitisinone is unknown. Understanding this mechanism could allow additional therapeutic approaches, crucial in managing patients with more advanced AKU disease. Pigment and pigment fragments have been described as being present in monocyte and monocyte‐related cells such as osteoclasts and osteocytes.^27^ Fisher and Davis studied an ochronotic femoral head ex vivo and showed that the pigment was not found in osteoblasts but was present in the calcified matrix as well as in the cytoplasmic vacuoles of osteoclasts and in osteocytes, some of which were degenerate or dead.^27^ HGA‐derived pigment has also been shown in macrophages.^28^ It is not convincingly proven at present whether monocyte/macrophage cells are involved in removal of ochronotic pigment, but is a potential mechanism for removal of pigment.

The conclusion that ochronosis reverses in the current data analysis differs from the finding in a mouse AKU model treated with nitisinone.^13^, ^14^ Only arrest of ochronosis was seen in the mouse post‐nitisinone, not a reversal. The reasons for the difference are not known. It is noteworthy that the mouse pigmentation is scanty compared to the human condition. Further, the mouse data related to pigment in the knee joint cartilage which is under constant loading, rather than the ear or the eye which may not be stressed to the same extent. We do not have an easy noninvasive way of studying internal ochronosis in loaded tissues at present. It is possible that Raman Spectroscopy could be adapted to study the Achilles tendon, a very common site of ochronosis in a highly stressed site. In addition, the pigment in the knee cartilage of the mouse is in the deep calcified articular cartilage, which might be less accessible to a mechanism that removes pigment.^13^, ^14^

The main limitation of the current analysis is that this was data collected as part of service provision. Therefore, since most patients were eligible for and were treated with nitisinone from the beginning, studying a no‐nitisinone AKU group has been challenging. The other main limitation is the lack of knowledge of the effect of nitisinone if any on internal ochronosis. Digital quantification of intensity and extent of pigment in photographs would be more powerful and reliable than semiquantitative categorical scoring employed here.

In conclusion, for the very first time in humans we have shown that ochronosis of AKU decreases significantly following HGA‐lowering by nitisinone therapy. This brings hope to those AKU patients with established disease that nitisinone therapy could reverse their disease to some extent.

### AUTHOR CONTRIBUTIONS

All authors contributed to analysis of the data, edited the manuscript, and approved the final version.

## ETHICS APPROVAL

The data collected from the NAC was approved by the Institutional Audit Committee (Audit No:ACO3836).

## CONFLICT OF INTEREST

The authors declare no conflicts of interest.

## Supporting information


**Table S1**. Scoring eye and ear ochronosis. *Eye pigmentation: 1, 2, and 3 points for slight, moderate and marked conjunctival pigmentation and 4, 6, and 8 points for scleral pigmentation; **Ear pigmentation: 2 and 4 points for slight and marked pigmentation.
**Table S2**. Ear biopsy scores at baseline V0 and 1 year after V3 (V4).
**Figure S1**. Tyrosine metabolic pathway—highlighting (1) the metabolic fate of tyrosine in health, (2) site of the enzyme defect observed in Alkaptonuria, *homogentisate 1,2‐dioxygenase* (*HGD* EC 1.13.11.5) and Hereditary Tyrosinaemia type 1, *fumarylacetoacetate hydrolase* (*FAH* EC 3.7.1.2), and (3) the site where nitisinone inhibits *4‐hydroxyphenylpyruvate dioxygenase* (*HPPD* EC 1.13.11.27) activity.
**Figure S2**. Scheme of visits to the NAC: The VAR group V‐1 visit consisted of the 10 patients from the SAME group plus 13 additional patients who attended the NAC twice without receiving nitisinone. The SAME refers to ten patients attending the research study between 2008 and 2011 (V‐1), followed by further annual visits to the NAC. The V0, V1, V2, and V3 refer to baseline and then three annual visits to the NAC. The numbers of patients in each group, their mean age, gender split (M = male; F = female), and years of follow‐up are also shown in the figure.
**Figure S3**. Eye and ear ochronosis scoring. *Eye pigmentation: 1, 2, and 3 points for slight, moderate and marked conjunctival pigmentation and 4, 6, and 8 points for scleral pigmentation; **Ear pigmentation: 2 and 4 points for slight and marked pigmentation.
**Figure S4**. Linear regression graphs of (A) sHGA and (B) uHGA_24_ against age. R refers to correlation coefficient. sHGA and uHGA_24_ are expressed as μmol/L and μmol/day, respectively.

## Data Availability

The authors agree to honor any reasonable request by other researchers for materials, methods or data necessary to verify the conclusion of the article.
