# Supplementary material for: Reversal of ochronotic pigmentation in alkaptonuria following nitisinone therapy: Analysis of data from the United Kingdom National Alkaptonuria Centre
Source: JIMD Rep. 2020 Jun 22;55(1):75–87. doi: 10.1002/jmd2.12137 (PMC7463057; doi:10.1002/jmd2.12137)
Supplement: Supplementary file 1 — Table S1. Scoring eye and ear ochronosis. *Eye pigmentation: 1, 2, and 3 points for slight, moderate and marked conjunctival pigmentation and 4, 6, and 8 points for scleral pigmentation; **Ear pigmentation: 2 and 4 points for slight and marked pigmentation. Table S2. Ear biopsy scores at baseline V0 and 1 year after V3 (V4). Figure S1. Tyrosine metabolic pathway—highlighting (1) the metabolic fate of tyrosine in health, (2) site of the enzyme defect observed in Alkaptonuria, homogentisate 1,2‐dioxygenase (HGD EC 1.13.11.5) and Hereditary Tyrosinaemia type 1, fumarylacetoacetate hydrolase (FAH EC 3.7.1.2), and (3) the site where nitisinone inhibits 4‐hydroxyphenylpyruvate dioxygenase (HPPD EC 1.13.11.27) activity. Figure S2. Scheme of visits to the NAC: The VAR group V‐1 visit consisted of the 10 patients from the SAME group plus 13 additional patients who attended the NAC twice without receiving nitisinone. The SAME refers to ten patients attending the research study between 2008 and 2011 (V‐1), followed by further annual visits to the NAC. The V0, V1, V2, and V3 refer to baseline and then three annual visits to the NAC. The numbers of patients in each group, their mean age, gender split (M = male; F = female), and years of follow‐up are also shown in the figure. Figure S3. Eye and ear ochronosis scoring. *Eye pigmentation: 1, 2, and 3 points for slight, moderate and marked conjunctival pigmentation and 4, 6, and 8 points for scleral pigmentation; **Ear pigmentation: 2 and 4 points for slight and marked pigmentation. Figure S4. Linear regression graphs of (A) sHGA and (B) uHGA24 against age. R refers to correlation coefficient. sHGA and uHGA24 are expressed as μmol/L and μmol/day, respectively. [file JMD2-55-75-s001.docx]

**Supplementary data**

Table S1

|  | | | |
| --- | --- | --- | --- |
| FEATURE | TEST | FEATURE | TEST |
| Eye ochronosis: | | | |
| Right eye nasal* | PHOTO | Left eye nasal* | PHOTO |
| Right eye temporal* | PHOTO | Left eye temporal* | PHOTO |
| Ear ochronosis: | | | |
| Right ear** | PHOTO | Left ear** | PHOTO |

Table S2.

|  | | |
| --- | --- | --- |
| Ear biopsy scores | V0 | V4 |
| Mean ± SD | 51.8 ± 25.9 | 41.9 ± 25.0 |
| n | 34 | |

Figure S1.


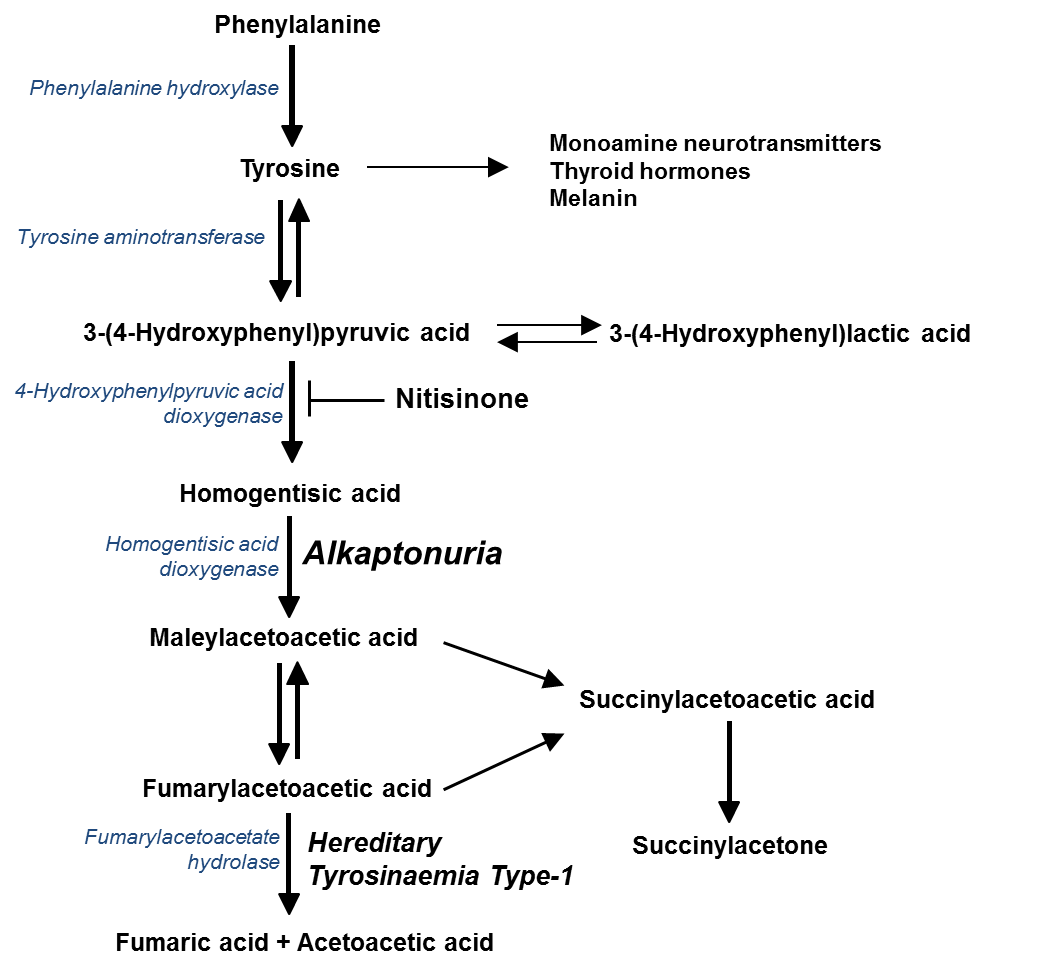


**Benzoquinone acetic acid**

**Ochronotic pigment**

Figure.S2

**n = 10 n = 10 n = 10 n = 10 n = 10**

**6 Male/4 Female**

**V3**

**V2**

**V1**

**V0**

**V-1**

**V-1**

**SAME GROUP**

**VAR GROUP**

**V3**

**V2**

**V1**

**V0**

PRE-NITISINONE

POST-NITISINONE

**Mean age 47.7±4.4 years**

**n = 23 n = 80 n = 52 n = 47 n = 40**

**Mean age 47.5±14.8 years 48.5±15.2 48.3±14.6 48.7±14.1 49.9±14.7**

**Gender split 15 Male/8 Female 50M/30F 32M/20F 29M/18F 24M/16F**

Figure S3.


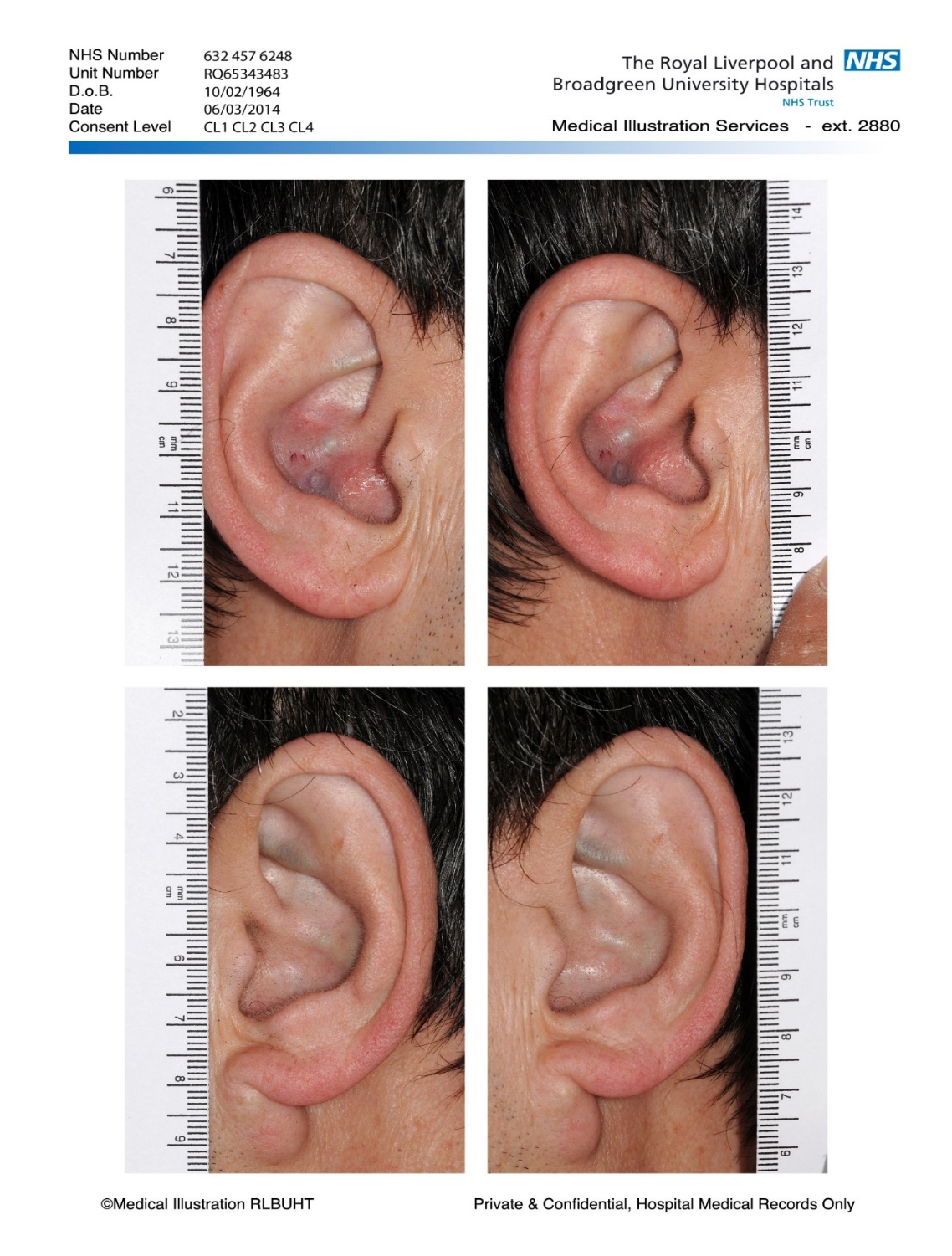

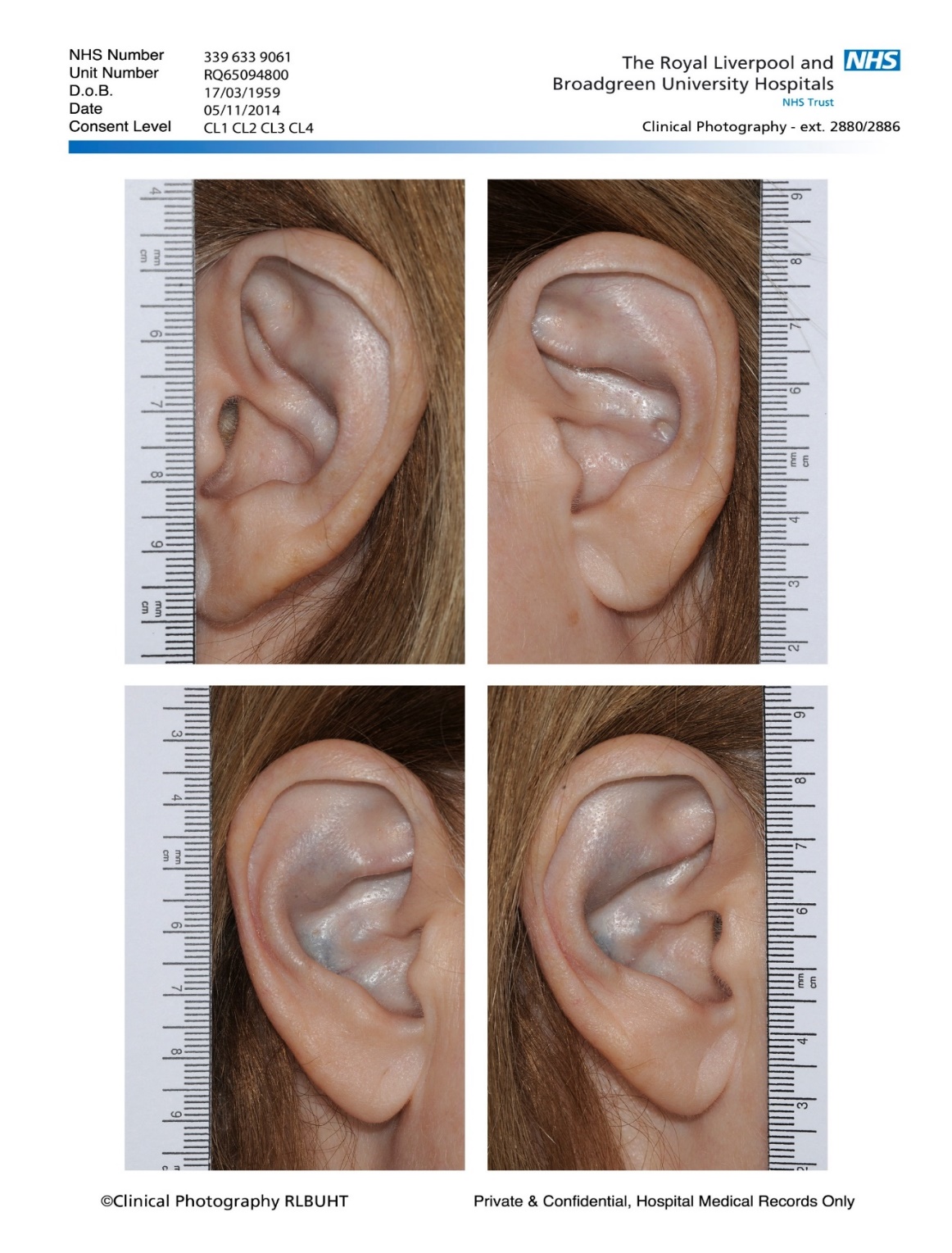


Present (2)

Marked (4)

**EAR SCORING**


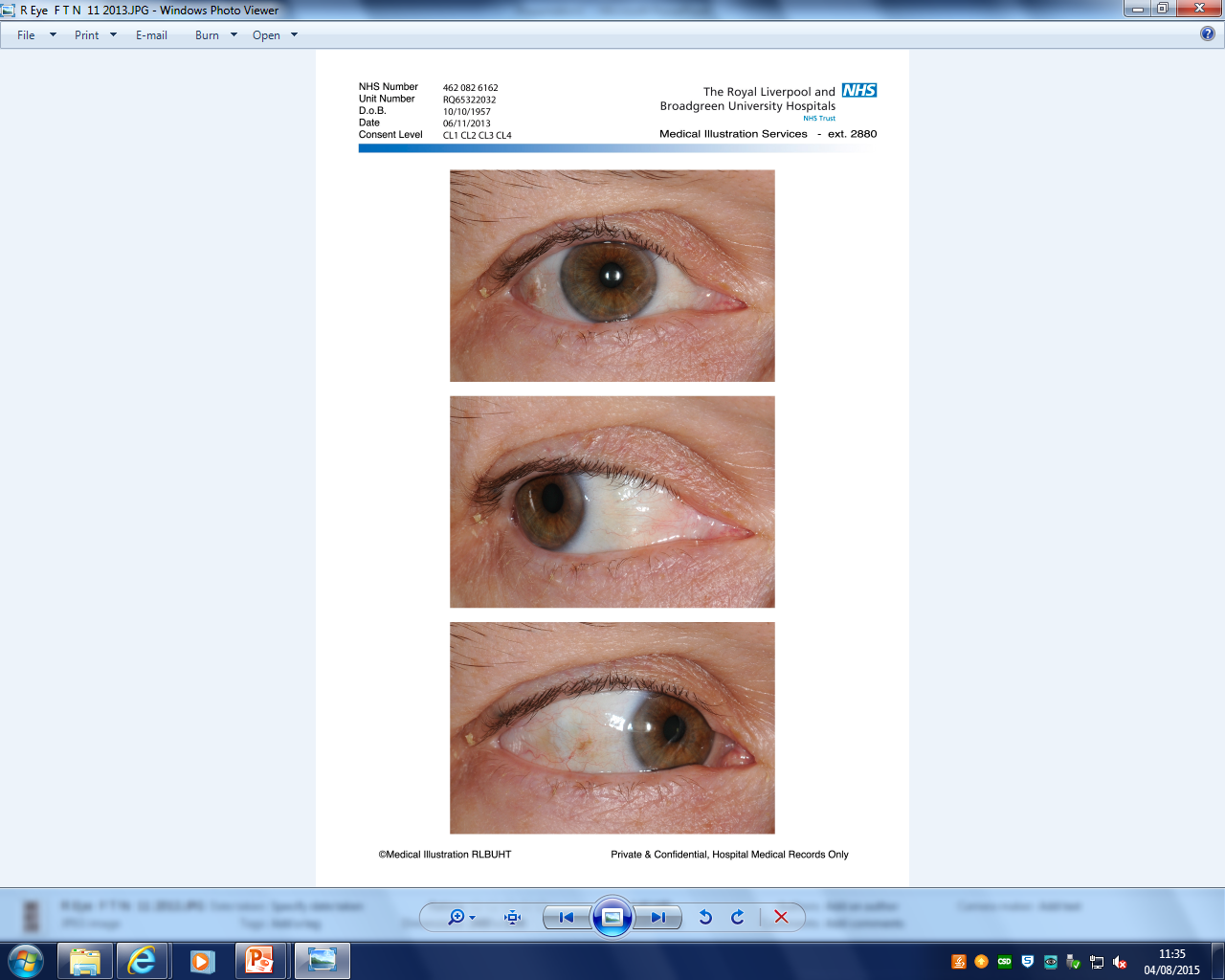

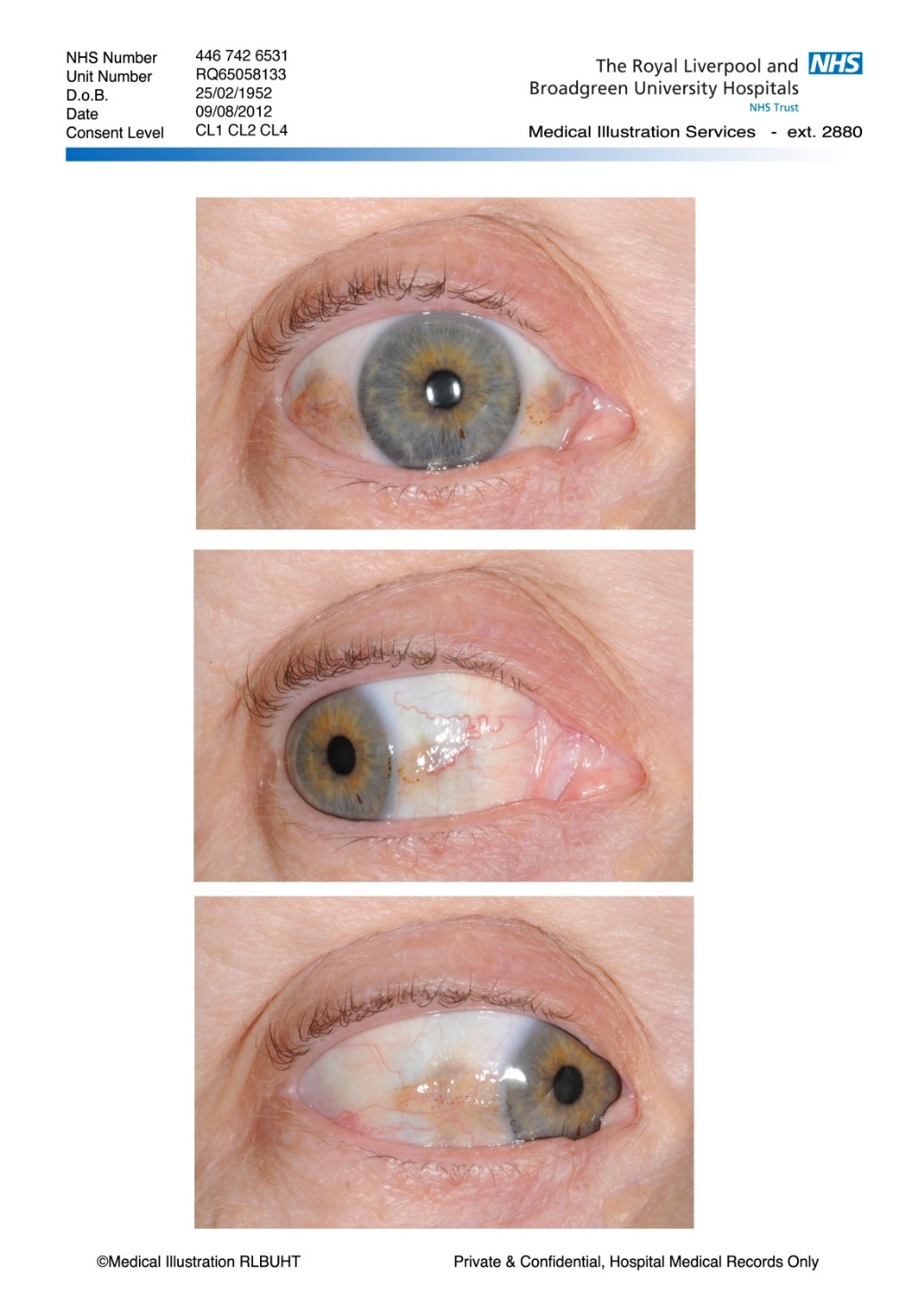

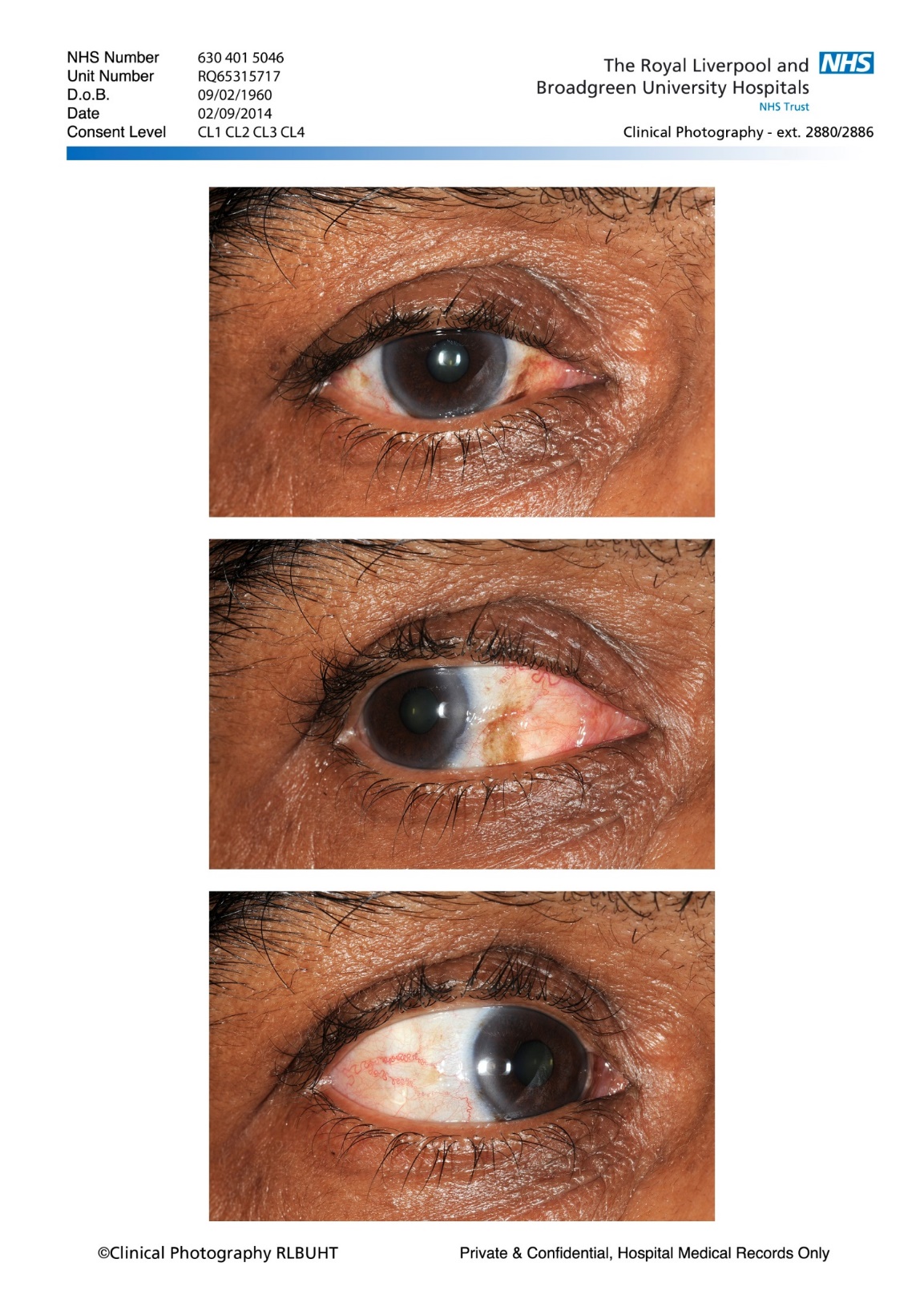


Mild (1)

Mod (2)

Marked (3)

SUPERFICIAL CONJUNCTIVAL PIGMENTATION


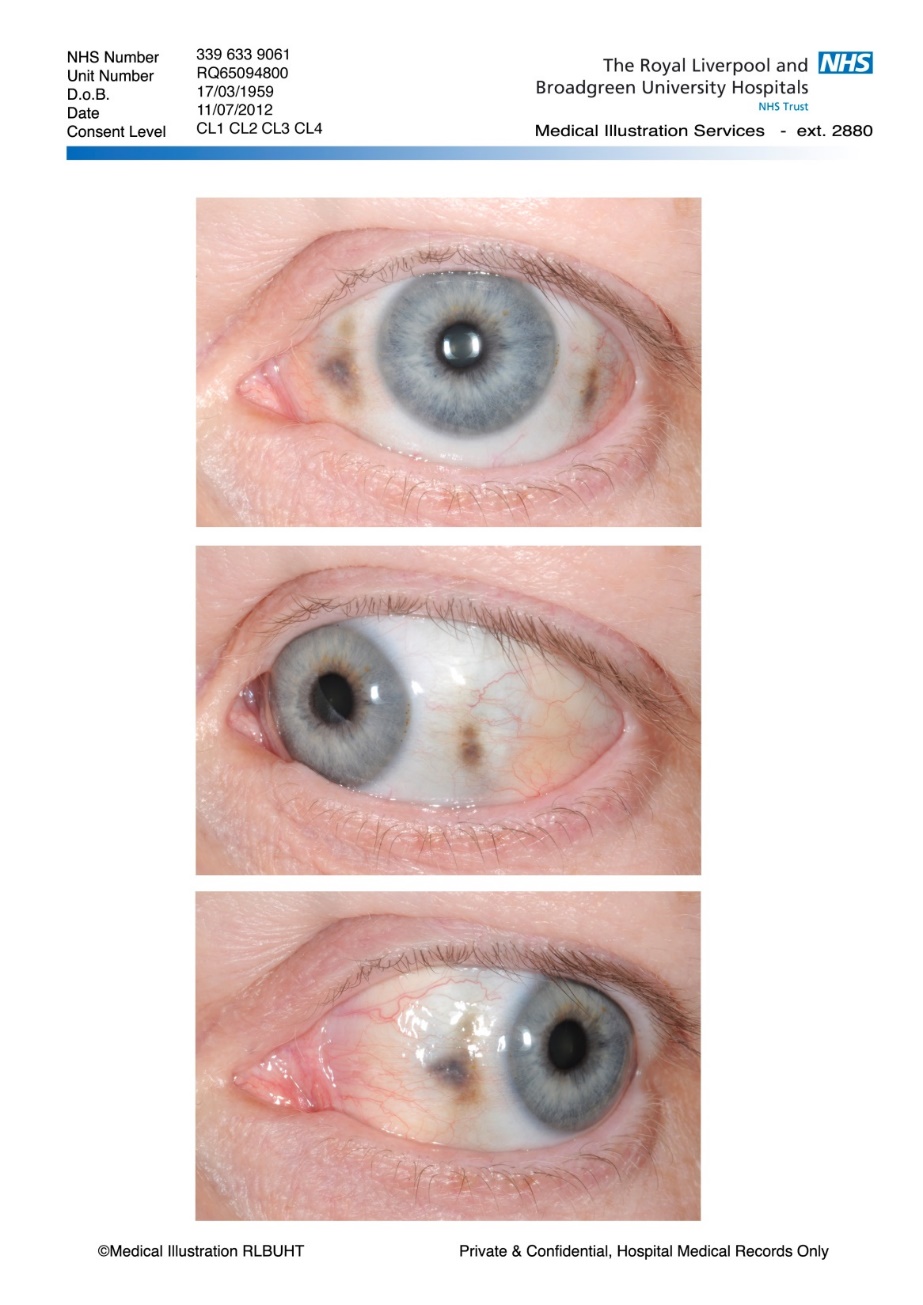

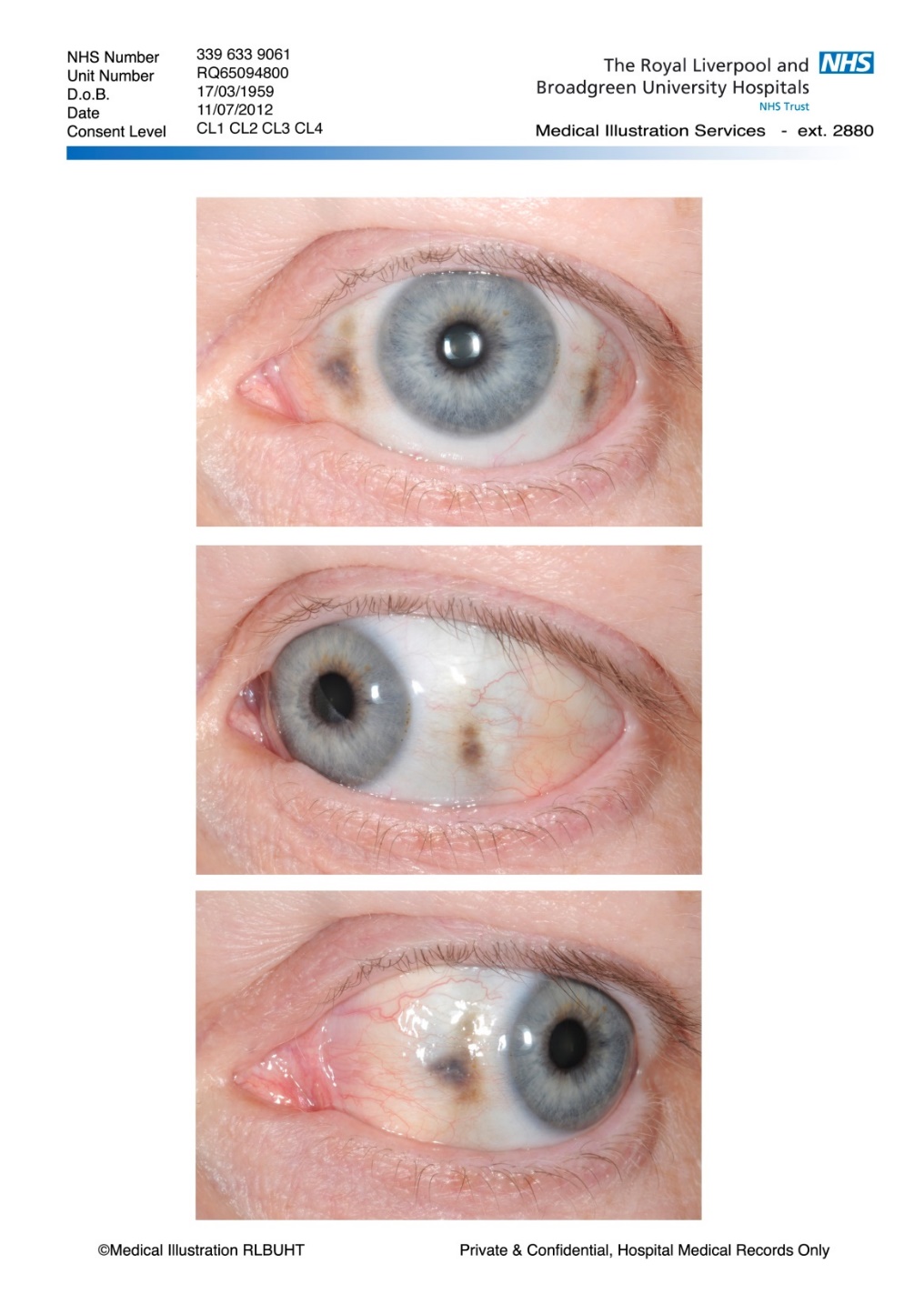

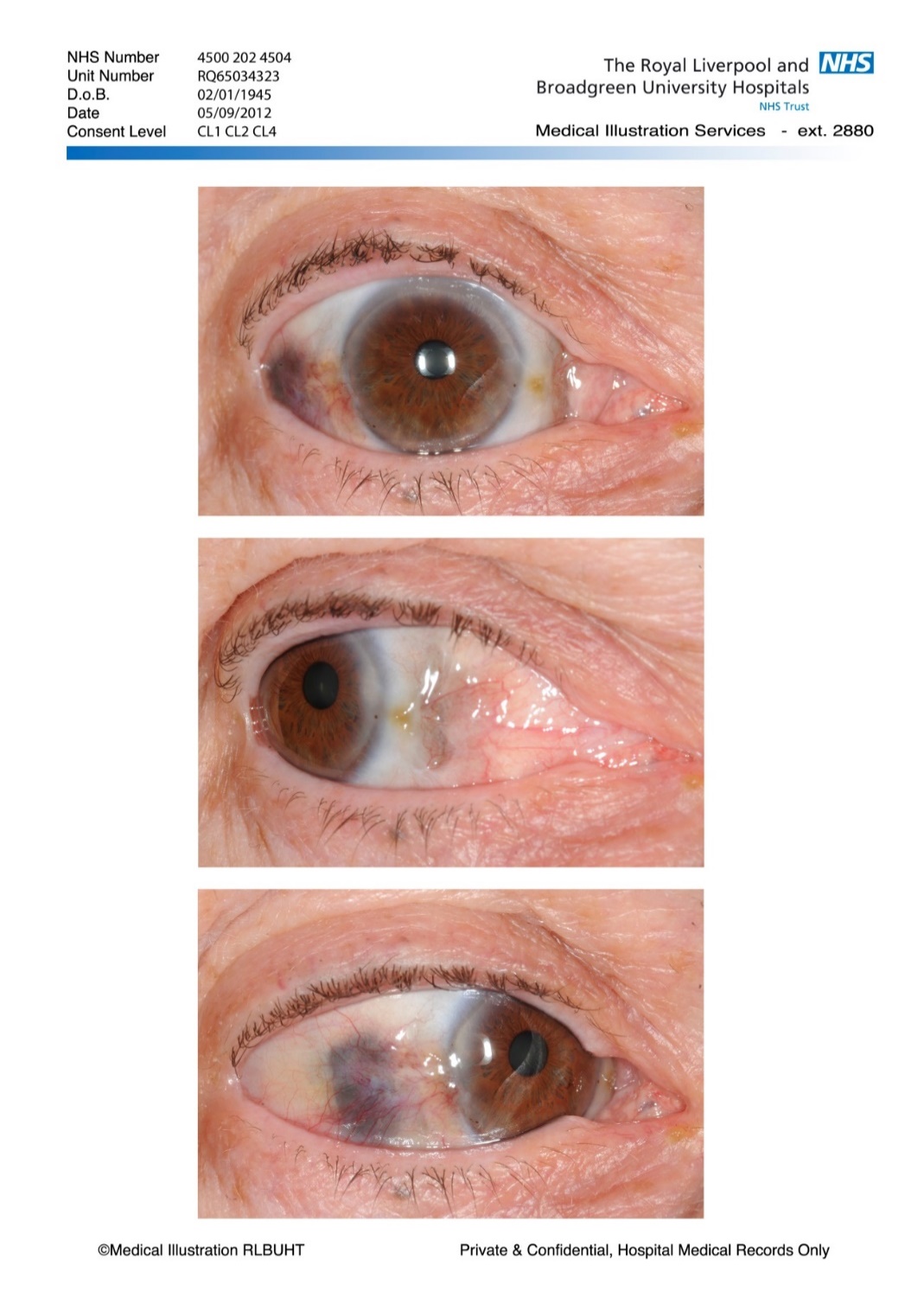


DEEPER SCLERAL PIGMENTATION

Mild (4)

Mod (6)

Marked (8)

**EYE SCORING**

**Figure S4.**

Age (years)


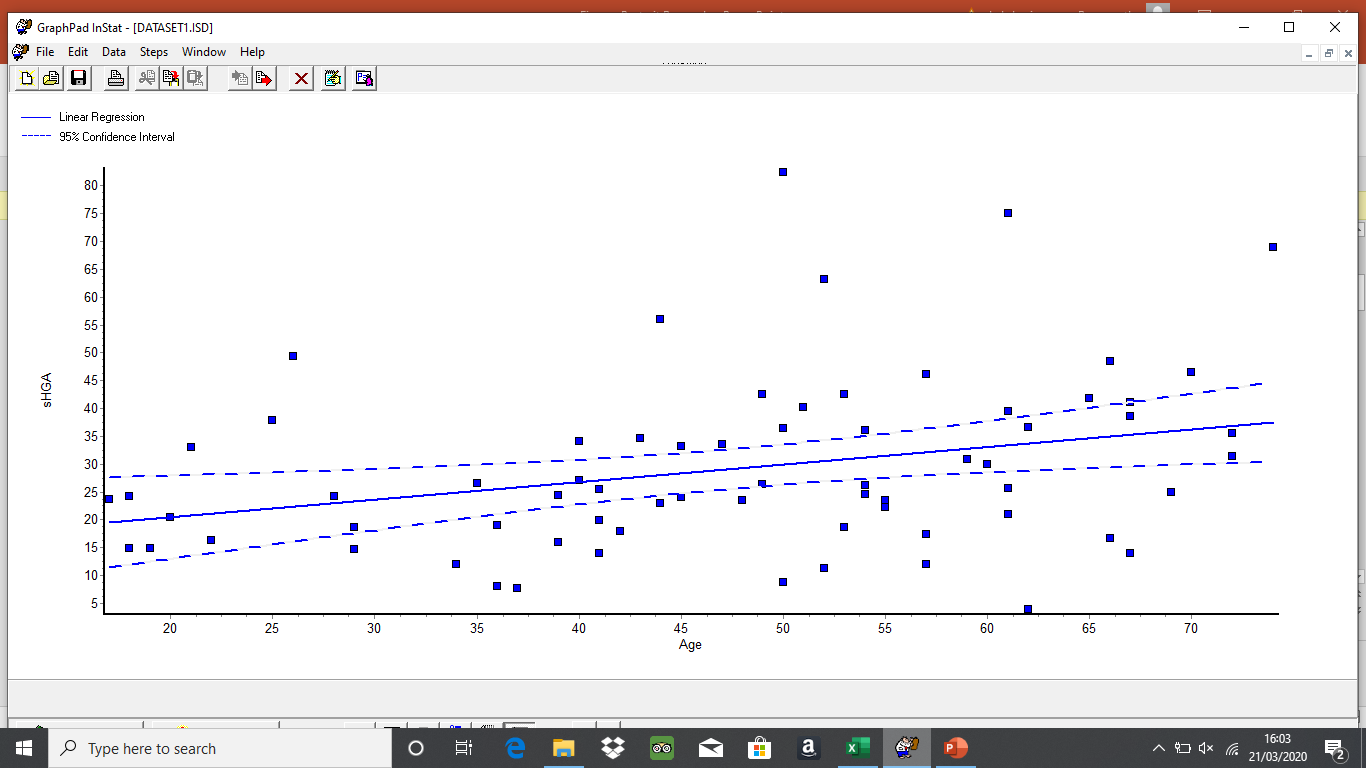


R = 0.31; p<0.01


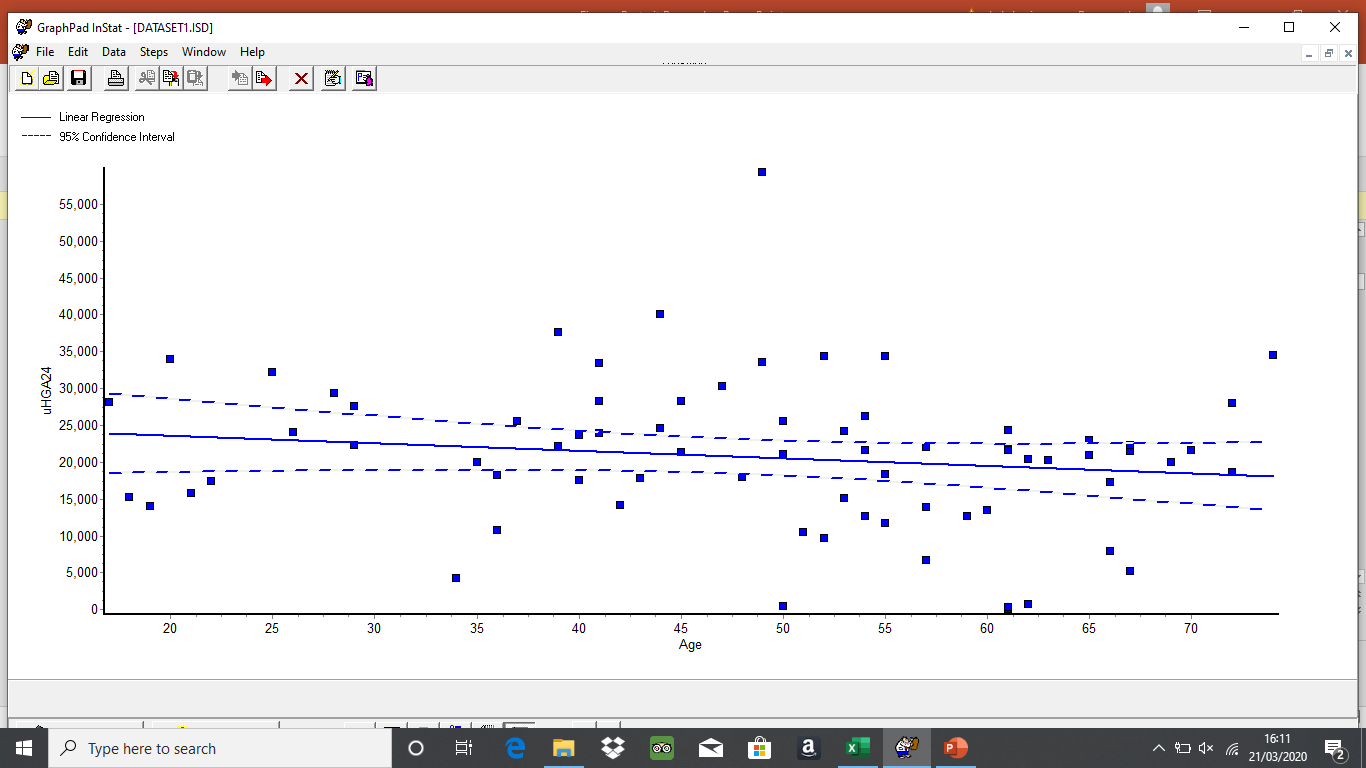


R = -0.16; p<0.19

B

A

Age (years)

Age (years)

uHGA_24_ µmol/day

sHGA µmol/l
